# Understanding the Adoption and Use of Digital Mental Health Apps Among College Students: Secondary Analysis of a National Survey

**DOI:** 10.2196/43942

**Published:** 2023-03-22

**Authors:** Justine Bautista, Stephen M Schueller

**Affiliations:** 1 School of Social Ecology University of California, Irvine Irvine, CA United States; 2 Department of Psychological Science University of California, Irvine Irvine, CA United States; 3 Department of Informatics University of California, Irvine Irvine, CA United States

**Keywords:** mental health, mental health apps, college students, digital health, app, anxiety

## Abstract

**Background:**

Increasing rates of mental health diagnoses in college students signal the need for new opportunities to support the mental health of this population. With many mental health apps being efficacious, they may be a promising resource for college campuses to provide support to their students. However, it is important to understand why (or why not) students might want to use apps and their desired features.

**Objective:**

Information on students’ interest in mental health apps may inform which apps are to be provided and how campuses can support their use. This study aimed to understand the interest and hesitation in app use and the relationship between mental health needs, as defined by depression, anxiety, and positive mental health, and app use.

**Methods:**

The web-based *Healthy Minds Study* collected information on mental health needs, perceptions, and service use across colleges and universities. We used a sample of 989 participants who completed the survey between 2018 and 2020 and an elective module on digital mental health. We analyzed the elective module responses using a mixed methods approach, including both descriptive and inferential statistics, along with thematic coding for open text responses.

**Results:**

The Results from this study revealed that anxiety (b=−0.07; *P*<.001), but not depression (b=0.03; *P*=.12) and positive mental health (b=−0.02; *P*=.17), was a significant predictor of app adoption. Prominent qualitative findings indicated that the most desired app features included tips and advice, access to resources and information, and on-demand support that involves interaction throughout the day. The participants also suggested an overall desire for human interaction to be integrated into an app. As predicted, hesitancy was encountered, and the qualitative results suggested that there was a lack of interest in the adoption of mental health app and preference.

**Conclusions:**

The findings from this study underscore that simply providing digital mental health apps as tools may be insufficient to support their use in college campuses. Although many students were open to using a mental health app, hesitation and uncertainty were common in the participant responses. Working with colleges and universities to increase digital literacy and provide resources that allow students to gauge when app use is appropriate may be helpful when implementing mental health apps as resources in college campuses.

## Introduction

### Background

From 2007 to 2017, the rate of mental health disorders increased from 22% to 36% among college students [1]. The most prevalent mental disorders in this group are depression and anxiety [2]. Compared with other health services offered on college campuses, mental health services have the highest number of visits per patient, indicating recurring service use and high demand for these services [3]. Increasing the number of traditional mental health services alone is unlikely to meet this demand. Many college campuses are facing budgetary challenges, making additional investments unlikely. Even when funding is made available, campuses face difficulties in filling open positions because of a shortage of qualified providers, especially those with experience in treating college student mental health disorders [4]. When services do exist, students may not use them because of their preferences or a need for care outside regular operating hours of services [5]. Most US counties suffer from a shortage of mental health providers, leaving college counseling centers with an unmet need and difficulty in recruitment [6]. With a shortage of mental health providers in general, colleges and universities must compete for a limited pool of candidates that are able to support college students and have the necessary qualifications [7], In light of this, campuses may need to explore new opportunities to support college student mental health. One opportunity includes digital mental health resources that can address this demand, with limited additional burden on traditional service providers.

### Mental Health App Engagement in College Students

Digital mental health is broadly defined as any form of technology used for mental health assessment, support, prevention, and treatment [8]. In particular, many campuses are offering digital mental health apps to their students either by purchasing or recommending them [9]. Digital mental health apps refer to software programs that provide tools and resources such as tracking, psychoeducation, and exercises to help people self-manage their conditions. Digital mental health apps are efficacious at addressing the mental health concerns of college students. Smartphone ownership is even higher in adults aged between 18 and 29 years (96%) than in adults (85%) generally [8]. Thus, college students may be an ideal population to target such resources. Because they can be accessed via mobile devices, mental health apps are widely available at any time, creating an especially accessible option for those who may have difficulty accessing mental health services. This is especially beneficial to college students, who may experience difficulty accessing campus resources when needed because of time constraints and staff shortages [5,7]. The convenience and flexibility of mental health apps may be especially appealing to college students. However, challenges in mental health app use include a lack of regulation of and research on many mental health apps available. With so many mental health apps being widely available, students may find it difficult to identify apps that contain evidence-based content, because research evidence for most mental health apps is scarce [9]. Furthermore, privacy and safety continue to be areas of concern, especially as privacy breaches continue to occur [9]. Nonetheless, several apps have been evaluated through prior research and deemed effective for alleviating mental health symptoms [10]. Thus, it is essential that college campuses provide safe, evidence-based, and effective tools for their students.

A systematic review of apps for college student mental health found that the majority of the apps evaluated were either effective or partially effective in alleviating symptoms of depression and anxiety or enhancing overall psychological well-being [10]. Despite their overall effectiveness, how best to get students to use mental health apps remains an open question. In light of this, more research is necessary to understand more specifically what college students may like or dislike in digital mental health apps. One study found that despite 53% of students downloading a digital mental health app at one point, only 19% were currently using a digital mental health app [11]. This signals a clear interest in using and downloading the apps, but a decrease in their actual use. In addition, most digital mental health interventions show low rates of adherence among college students, signaling a lack of sustained app use [12]. Nonetheless, those with clinical diagnoses of depression and anxiety seem to show increased interest in mental health apps [13]. In particular, web-based health resource use is especially high in those with mild to severe symptoms of depression [14]. The existing interest in those with anxiety and depression combined with their clear efficacy in alleviating symptoms leads us to believe that anxiety and depression may have an association with app use. However, low rates of adherence have also led us to explore whether this interest in apps translates to their actual use.

This study seeks to bridge the gap between interest in apps and app use, exploring why college students may choose to use an app or not. Specifically, we explored college students’ existing attitudes toward mental health apps and the relationship between mental health needs, including depression, anxiety, and positive mental health, and app use. We hypothesized that we would see both positive and negative themes in attitudes with respect to mental health apps and that apps would be generally well received but would continue to encounter attitudes of uncertainty about their efficacy. We also hypothesized that depression and anxiety would have a significant association with mental health app adoption and the frequency of app use. Few studies have examined the association between positive mental health and app use; nevertheless, we hypothesized that positive mental health would not be a significant predictor, as no concrete link between the 2 variables has been identified in the past.

## Methods

### The Healthy Minds Study

This study used data from the *Healthy Minds Study,* an annual web-based survey distributed to colleges and universities across the United States. The *Healthy Minds Study* addresses various topics related to college student mental health, campus climate, help-seeking, service use, and overall student experience [15]. Incentives included a national sweepstakes, in which 2 students were selected for US $500 gift cards, while 10 were selected for US $100 gift cards [16]. However, institutions may also provide their own incentives of their choice, with approval from the Healthy Minds Study team [16].

The survey is structured in 2 parts: standard modules and elective modules. Each participating college or university is required to administer the standard modules, consisting of basic demographic information (age, sex, race and ethnicity, socioeconomic status, etc), academic information, school experiences, mental health status, and mental health service use or help-seeking. Colleges and universities are also provided with a list of elective modules on topics such as substance use, sleep, eating and body image, sexual assault, overall health, and financial stress. Each college or university may select which of these elective modules are to be provided along with the standard modules. Thus, each student completing the survey receives the combination of standard modules and the university- or college-selected elected modules. Our analysis focuses on data collected from 2018 to 2020 after *Attitudes About Mobile Resources* elective module was introduced. However, the exact dates of data collection were unavailable in the data set provided.

### Ethics Approval

Institutional Review Board (IRB) approval for the Healthy Minds Study was obtained through Advarra, an independent IRB service in North America. This study makes use of the publicly available and deidentified Healthy Minds Study data that are made available through this website [17]. This secondary data analysis was deemed exempt from an additional IRB review by the University of California, Irvine.

### Participants

Participants consisted of college students who completed the *Healthy Minds Study* survey from 2018 to 2020. A broad range of colleges and universities participated in this study, with students pursuing an associate’s degree, bachelor’s degree, master’s degree, JD, MD, PhD, or equivalent, as well as nondegree students being included in this sample. Overall, the data set consisted of 151,211 participants, of which 62,029 (41.02%) participants were from 78 colleges and universities during the 2018 to 2019 academic year, and 89,182 (58.98%) participants were from 75 colleges and universities during the 2019 to 2020 academic year. The 2018 to 2019 survey had a 16% participation rate while in the 2019 to 2020 academic year; the participation rate was 13% in the fall and 16% in the winter. Participation rate data were provided by The Healthy Minds Network. The 2018 to 2019 survey did not differentiate between fall and winter survey participation rates. Only a subset of participants completed our module of interest, “Attitudes About Mobile Resources.” In the 2018 to 2019 academic year, 400 (0.64%) participants completed the module, whereas 589 (0.66%) participants completed the module in the 2019 to 2020 academic year, resulting in a total of 989 participants.

Overall, participants who completed the elective module were relatively similar to the broader sample. Both the samples had participants who were on an average aged 23 (SD 4.92) years; mostly female (overall: 101,000/151,211, 66.79%; elective module: 673/961, 70%); and mostly White (overall: 107,003/151,211, 70.8%; elective module: 788/961, 82%). Those who completed the elective module and the overall sample both had an average score of 44 (8.12) on the Diener and Diener Flourishing Scale but the sample that completed the elective module had slightly elevated percentages of students with depression (overall: 45,193/151,211, 29.89%; elective module: 310/961, 32.3%) and anxiety (overall: 39,126/151,211, 25.87%; elective module: 274/911, 28.5%). Furthermore, those who completed the elective module tended to pursue higher levels of education. Fewer students who completed the elective module were pursuing an associate’s degree (overall: 14,150/151,211, 9.36%; elective module: 14/961, 1.6%), while a higher percentage of students were pursuing a bachelor’s degree (overall: 100,548/151,211, 66.49%; elective module: 611/961, 69.1%); master’s degree (overall: 18,325/151,211, 12.12%; elective module: 179/961, 20.2%); JD (overall: 980/151,211, 0.65%; elective module: 11/961, 1.2%); MD (overall: 1926/151,211, 1.27%; elective module: 17/961, 1.9%); or PhD (overall: 10,683/151,211, 7.06%; elective module: 130/961, 14.7%).

### Measures

From the standard module, we used 3 measures to identify the mental health status of the participants. Depression was measured using the Patient Health Questionnaire-9 (PHQ-9), anxiety using the Generalized Anxiety Disorder Scale-7 (GAD-7), and positive mental health using the Diener and Diener Flourishing Scale.

*The PHQ-9* is a 9-item measure of depressive symptoms [18]. PHQ-9 total scores range from 0 to 27, with a PHQ-9 score of >9 indicating clinically elevated levels of depressive symptoms [18]. The psychometric properties of the PHQ-9 evaluated in college students demonstrated its reliability and validity, even in diverse college student populations [19].

*The GAD-7* contains 7 items measuring generalized anxiety [20]. GAD-7 total scores range from 0 to 21 [20]. A GAD-7 score of >9 indicates clinically elevated levels of anxiety symptoms [20]. Prior analyses of the GAD-7 suggested that it has strong reliability and validity when used as a screener for college students [21].

*The Flourishing Scale* is an 8-item scale measuring self-perceived success in various important areas of a participant’s life [15]. Higher overall scores on the Flourishing Scale indicate greater psychological resources, with scores ranging from 8 to 56 [22]. The Flourishing Scale has demonstrated high reliability in measuring the well-being among university students [23].

*The “Attitudes About Mobile Resources”* elective module included the following questions:

1. “Would you be open to using an app for wellness or mental/emotional health?” (Yes; maybe; no) “Have you ever used a smartphone app to manage your wellness or mental/emotional health?” (No, never; yes)
2. “When did you use a smartphone app to manage your wellness or mental/emotional health?” (Before starting college; since starting college; I currently use an app)
3. “What are the reasons why you have not used a mental health app?” (I have concerns about privacy and security of data; there is lack of research support available; I’m unsure about how useful the app will be; I have concerns about cost; apps seem difficult to use; I don’t know if I could find a suitable app; I don’t know which app to download; I don’t have time to use apps; I’m not interested in using mental health apps; I don’t think I need these kinds of apps; Other)
4. “Now imagine you are trying to decide which wellness or mental/emotional health app to use. How important would each of the following features be in your decision? (The app has research supporting its benefits; the app is well designed and easy to use; the app has information about data privacy and storage policies; the app has reviews from users; the app has reviews from experts in the field; the app developer; the cost of the app; the time commitment required by the app seems manageable to me; Something else).” (Not at all important; slightly important; moderately important; important; very important)
5. “How helpful, overall, do you think the smartphone app(s) was or has been for your mental or emotional health?” (Very helpful; helpful; somewhat helpful; not helpful)
6. “What would you hope to get out of an app for wellness or mental/emotional health?”
7. “Why would you not use a mental health app?”

### Data Analysis

This study used a mixed methods approach, drawing from both qualitative and quantitative methods. Quantitative methods included both descriptive and inferential statistics to assess the variables that may affect app adoption. Qualitative methods included thematic analyses of open text responses to questions related to app use. The qualitative methods were intended to expand the findings and understand the quantitative responses.

### Quantitative Methods

We aimed to understand the relationship between mental health (as measured by PHQ-9, GAD-7, and Flourishing scale) and mental health app use (adoption and frequency). To do so, we conducted a binary logistic regression with mental health status predicting adoption while controlling for demographic variables, including race and ethnicity, sex, age, and socioeconomic status. Independent binary logistic regressions were conducted for each mental health status variable (ie, depression, anxiety, and flourishing). Logistic regression was used to examine the relationship between mental health and the frequency of app use. To determine if clinical levels of mental health symptoms were related to app use, chi-squared difference tests were conducted with clinical thresholds identified by the PHQ-9 and GAD-7 (>9 for both measures). As we were simultaneously conducting statistical tests for separate mental health variables, Bonferroni correction was used to adjust the α level and address multiple comparisons. The adjusted α level was .017. Raw percentages were also calculated for the number of students who had ever used a mental health app and who would or would not be open to using a mental health app, the reasons for hesitation in app use, and the time points when students initiated app use.

### Qualitative Methods

A thematic analysis [23] was conducted on the 2 open text responses in the elective module, and not all students answered each question. The response totals are reported below.

1. “What would you hope to get out of an app for wellness or mental/emotional health?” (541 responses)
2. “Why would you not use a mental health app?” (131 responses)

We used open coding to identify themes in attitudes toward mental health apps and their use. We created the codebook by first performing an initial analysis of the responses from all participants and then revising the codebook iteratively after an initial subset of the data was coded. After coding 10% of the data, the coders reviewed the responses and identified areas where more clarity was needed in the codebook. The 2 coders then coded the next 10% and continued to revise the codebook. Coding in segments also promoted increased reliability and consistency, allowing the 2 coders to review discrepancies and areas where the codebook could be refined. After coding 20% of the data, discussing, and revising the codebook, coders independently completed the remainder of the responses. In the final codebook, question 1 had a total of 38 codes, whereas question 2 had a total of 12 codes. We assessed the interrater reliability with percent agreement, which was 80.3% for question 1 and 91.2% for question 2. Given the high level of agreement and the goal to identify themes present in the data, rather than requiring consensus, a code was counted as present if either of the coders indicated that code for a particular response. Thus, some responses received 2 codes when the coders were not in agreement.

## Results

### Demographic Information

A total of 961 students completed the elective module. These 961 students were predominantly White (n=788, 82%) and female (n=673, 70%). The average age of the participants was 22.63 (SD 4.92) years. The mean level of the mental health variables was 7.98 (SD 5.94) for depression, 6.98 (SD 5.38) for anxiety, and 44.03 (SD 8.12) for positive mental health. The rates of clinically elevated depression and anxiety levels were 32.3% (310/961) and 28.5% (274/961), respectively.

### Quantitative Results

Table 1 presents the percentages of app use and the reasons for hesitation across the sample. The results of the binary logistic regression for depression, anxiety, and positive mental health as predictors of app use found that after controlling for sex, age, socioeconomic status, and race and ethnicity, anxiety was the only significant predictor of app adoption (b=−0.07; *P*<.001). Neither depression (b=0.03; *P*=.12) nor positive mental health (b=−0.02; *P*=.17) were significant predictors of app adoption. We used McFadden pseudo-*R*^2^ (*ρ*^2^) to calculate goodness of fit (*ρ*^2^=0.03), which indicated a small effect size.

We also examined whether depression, anxiety, and positive mental health would predict greater frequency of app use. The resulting model did not show a significant relationship between the mental health variables and frequency of app use (*R*^2^=0.05; *F*_13,230_=0.9; *P=*.56). Investigation of the coefficients showed that the presence of depression (b=0.02; *P*=.63), anxiety (b=−0.003; *P*=.91), and positive mental health (b=0.01; *P*=.57) had no significant relationship with the frequency of app use. We used a chi-squared test of independence to analyze whether those who met the clinical thresholds of depression and anxiety showed higher rates of app adoption. No relationship was found between anxiety (*χ*^2^_1_=2.8, N=961; *P*=.10) and app adoption and depression (*χ*^2^_1_=0.9, N=961; *P*=.34) and app adoption.

**Table 1:** App use and hesitation.

Response			Values, n (%)
**Students who have used a mental health app**			
	Yes	245 (25.4)	
	No	719 (74.6)	
**Students who would be open to using a mental health app**			
	Yes	368 (38.02)	
	No	144 (14.88)	
	Maybe	456 (47.11)	
**Time points when students used a mental health app**			
	Before starting college	64 (26.1)	
	Since starting college	172 (70.2)	
	Currently using	85 (34.7)	
**Hesitation to use a mental health app**			
	I’m unsure about how useful the app will be	351 (48.8)	
	I don’t know which app to download	290 (40.3)	
	I don’t need these kinds of apps	262 (36.4)	
	I don’t know if I could find a suitable app	193 (26.8)	
	I’m not interested in using mental health apps	191 (26.6)	
	I have concerns about privacy and security of data	139 (19.3)	
	I don’t have time to use apps	101 (14.1)	
	I have concerns about cost	82 (11.4)	
	I don’t have a suitable device or enough space to download apps	34 (4.7)	
	There is a lack of research support available	68 (9.5)	
	Apps seem difficult to use	15 (2.1)	

### Qualitative Results

The first question that we qualitatively analyzed asked students what they would like to see in a mental health app. We grouped the codes into categories as shown in Table 2. The first was “what they want” and the second was “how they want it.” The “what they want” category consisted of codes that addressed specific app features. Upon calculating the frequency of the codes, we found that the 3 features that students were most interested in were related to tips and advice. The third most prominent code was also closely related to this, with many asking for “relaxation/calming tips,” which refer to apps that may have features for calming or reducing stress in the user. Some examples of student responses within this theme are “Tips and tools for when feeling anxious...” and “Tips/strategies for dealing with stressors in my life.”

“Access to resources and information” was also a prominent code. Example responses included, “information to read, resources listed” and “...find in-person resources.” Another larger proportion of code seemed to suggest that students want on-demand support that involved interaction throughout the day. Students wanted “tracking and documenting/journaling” features along with “reminders and check-ins.” Example responses include, “tracking factors that contribute to emotional wellbeing” and “reminders of how to not be anxious or deal with situations, or just reminders.”

**Table 2:** Desired mental health app features.

Code					Values, n (%)
**What they want**					
	Tips and advice (general)			90 (16.7)	
	Anxiety and mental wellness tips			68 (12.6)	
	Relaxation or calming tips			58 (10.8)	
	Not sure or I don’t know			58 (10.8)	
	Access to resources and information			57 (10.6)	
	Tracking and documenting or journaling			52 (9.6)	
	Reminders and check-Ins			42 (7.8)	
	Guided meditation			41 (7.6)	
	Access to medical help and appointments			30 (5.6)	
	Encouragement and Motivation			20 (3.7)	
	Daily tasks or routines and goal-setting			18 (3.3)	
	Positive affirmations			16 (3)	
	Holistic well-being			15 (2.8)	
	Activities			13 (2.4)	
	An emotional outlet			13 (2.4)	
	Confidence and empowerment			12 (2.2)	
	Breathing exercises			11 (2)	
	Health and physical exercise tips			10 (1.9)	
	Productivity and time management			9 (1.7)	
	Clarity and perspective			8 (1.5)	
	Comfort			8 (1.5)	
	Stability			8 (1.5)	
	Depression help			4 (0.7)	
	Self-care tips			3 (0.6)	
	Sleeping tips			3 (0.6)	
	Help with suicidal thoughts			1 (0.2)	
**How they want it**					
	**Communication and real-time support**			68 (12.6)	
		Professional support	24 (4.5)		
		Peer support	23 (4.3)		
	Convenience in accessibility			23 (4.3)	
	Affordability			7 (1.3)	
	Anonymity			7 (1.3)	
	Similarity to other apps			7 (1.3)	
	Usability			6 (1.1)	
	Confidentiality			5 (0.9)	
	Unique to existing resources			4 (0.7)	
	No judgment			3 (0.6)	
**Hesitation in using mental health apps**					
	Not helpful, ineffective, or not useful			29 (22.3)	
	I don’t need it			25 (19.2)	
	I would not use it			25 (19.2)	
	Prefers face-to-face counseling or in-person interaction			16 (12.3)	
	Privacy and confidentiality concerns			14 (10.8)	
	Dislike for apps			11 (9.2)	
	Smartphone worsens mental health			12 (8.5)	
	Impersonal			8 (6.2)	
	Already using a mental health app			5 (3.8)	
	I don’t own or have limited access to a smartphone			4 (3.1)	
	Not sure or I don’t know			4 (3.1)	
	Religious and family support			3 (2.3)	

In the “how they want it” category, students showed an overwhelming desire for “communication and real-time support” features. This code had 2 subcodes, “professional support” and “peer support,” which were both prominent codes in our analysis. Example responses include, “An anonymous peer-peer chat group or access to a mental health professional” and “convenience of a mental health professional anywhere and anytime.” This suggests that one theme of these responses is the overall desire for human interaction to be integrated into an app. Students wanted the ability to communicate in real time with both peers and professionals.

Our second question aimed to explore reasons why students may not use a mental health app, providing insight into hesitation in mental health app adoption. The most prominent responses were coded as “not helpful/ineffective/not useful.” “I don’t need it” and “I would not use it” were also prominent codes, which reflects a lack of interest. Example responses include, “i don’t think talking to a computer could help,” “worried about the ability of such an app,” and “I do not think that it could provide realistic or helpful information.” These 3 responses may provide insight into how resources in college and university settings may not be well received by some students. In addition, the responses indicated a preference for face-to-face counseling or in-person interaction, adhering to more traditional methods of mental health help-seeking.

## Discussion

### Principal Findings

Our findings suggest that simply providing digital mental health apps may not be sufficient to increase their use among college students. Rather, we identified considerable hesitation and uncertainty toward using mental health apps and the various factors related to app adoption. Despite numerous studies examining perceptions and outcomes of mental health app use, few studies give college students the space to openly discuss and identify desired individual app features, hesitation, and barriers to app use. Our findings provide information on how college students use mental health apps, their interest in particular app features or capabilities, and hesitation that may affect app adoption.

Contrary to our hypothesis, which suggested that anxiety and depression would both be significant predictors of app adoption, our quantitative findings suggested that anxiety was the only significant predictor of app adoption. This aligns with our qualitative findings in which one prominent feature that students wanted in mental health apps was tips and advice related to anxiety and relaxation. Students wanted apps that directly addressed how to reduce anxiety symptoms and promote relaxation. Prior literature suggests that meditation apps are among the most commonly downloaded mental health apps, and meditation is largely popular among those with anxiety [24,25]. With meditation apps being popular and appealing to those with anxiety, college campuses should consider them as a potentially valuable resource. Surprisingly, the qualitative analysis also found very few responses indicating that the students wanted to see features that directly addressed depression. This could be due in part to meditation apps, with meditation appealing to those with anxiety and being the most popular category of mental health apps and the most well known. Those with anxiety may feel that the most popular mental health apps are more tailored to their needs than those with depression. Regarding positive mental health, our findings also suggested that it did not affect app use. One explanation for these findings is that negative emotions, and the desire to reduce them, might drive the adoption of mental health apps among college students rather than positive emotions. Some conceptualizations of depression and anxiety characterize them by the presence of positive and negative emotions [26]. In such conceptualizations, depression is characterized by low positive affect and low negative affect, whereas anxiety is characterized by low positive affect and high negative affect. This would be consistent with the lack of significant findings for positive mental health, which would be thought of as consisting of high positive and low negative affect [27].

It should also be noted that only about 25% (245/961) of the respondents reported using a mental health app. This is in contrast to prior literature, which suggests that 53% of college students have used a mental health app [28]. Much of the prior work, however, has focused on 4-year universities, and our data set consisted of a wide variety of colleges and universities. Indeed, one study of community college students found that 21.2% reported using a mental health app [29]. Thus, adoption rates may vary across types of colleges and universities, and more work should be conducted that is more representative of different types of higher educational settings.

Although anxiety predicted app adoption, it did not predict more frequent app use. One explanation would be that stress may inhibit sustained mental health app use [11]. As college students face stressors associated with academics, financial issues, and peer relationships, using a mental health app may not fit into their already busy lives. This may also be due to the uncertainty surrounding app use and perhaps a lack of digital literacy regarding the use of an app. Students may be unsure of how to use the app or identify how often to use it. This could explain why students with elevated anxiety are not likely to use a mental health app more frequently, although they are more likely to download a digital mental health app. Even if a student with anxiety were to download a mental health app, they may be unable to identify how often they should use it based on their symptoms. In contrast, it is also possible that anxiety did not predict frequent app use because those who used apps frequently experienced reduced anxiety. Given that our data were cross-sectional, we could not determine how adoption might lead to changes in clinical outcomes and, ultimately, impact long-term use.

With other methods, such as traditional in-person therapy or prescribed medications, patients receive direct instruction and are given a schedule for therapy sessions or medication dosage. When using a mental health app, students must independently decide what a “healthy” amount of app use looks like and what apps would be useful in relation to their symptoms. The second largest point of hesitation was uncertainty about which apps they should download. Increasing digital literacy and providing tools that allow students to gauge when app use is appropriate may be helpful when implementing digital mental health apps as resources in college campuses. Working with ecosystems that already exist in students’ lives may be especially beneficial. For example, Kaiser Permanente created a set of mental health apps to provide to their members. To support delivery and integration, health care providers within the Kaiser Permanente network were trained to provide a web-based mental health tool as a resource [30]. Similar efforts could benefit college campuses by training counseling center staff or other providers, peers, or students to increase digital literacy and find effective evidence-based mental health apps. Digital navigators, or people designated to evaluate and recommend apps, set up technology, and collect data within the organization, can work with counseling center staff to implement mental health apps on campus [31]. In addition, factors that predict a student’s decision to begin using an app may not predict their sustained use. It is possible that those with higher anxiety levels did not experience sufficient improvement to continue using the app. However, it is also important to note that these data were cross-sectional. Consequently, it is difficult to determine the full scope of discovery, adoption, and sustainment.

In students who had used a mental health app since starting college, only about half were still using one at the time the survey was completed. In line with findings from the study by Kern et al [32], students have a clear interest in mental health apps but do not sustain their use. Again, it is possible that as students experience better mental health, the need for a mental health app no longer exists, leading them to delete the app. However, it is also possible that uncertainty around app use may prevent students from using an app or continuing its use.

In addition, it is not surprising that an overarching theme was that students wanted apps to provide resources and increased access to mental health care. Access to resources and information along with access to medical help and appointments ranked highly on the list of the desired app features. The most common theme for the “how they want it” section suggested that students wanted apps that provided real-time support and connected them with both peers and professionals. Most mental health apps seem to have similar features, with mood tracking and journaling being the most common [33]. Apps that connect students to resources and increase access to medical care, professional support, and peer support may be worthwhile for this population. However, none of these features are mentioned in the list of the top 5 most common offerings of mental health apps [31]. In the study by Lagan et al [33], the analysis revealed that very few apps offered peer support and connection to a coach or therapist, whereas none offered access to resources and information or access to medical care. In addition, no themes emerged regarding cultural competency, although our sample consisted of White individuals predominantly. Integrating campus resources with mental health apps may be of particular interest to college students [34]. Fortunately, there are some products that demonstrate these features. For example, YOU at College is a “full continuum of campus care,” and it provides multiple features. Their “YOU” app connects students to resources available on their campus along with other resources and content related to mental health. In addition, their “NOD” app is a cognitive behavior therapy–based app that provides social challenges, prompts, and reflection opportunities to combat loneliness and avoidance. The “NOD” app is built on evidence-based principles and has been shown to reduce loneliness in susceptible students [35]. Resources such as these may be useful for campuses to consider, especially as services, such as YOU at College require enterprise adoption, that is, a college or university must purchase it to make it available for its students.

In students who had not used a mental health app, a number of factors led to hesitancy. It was most common for students to indicate that they were unsure of how useful the app would be. The second major point of hesitation was the uncertainty around which app to download. With so many apps existing in the app store, it is understandable why it might be difficult for students to select one. The app store houses thousands of mental health apps, with very few actually having empirical support [36]. Therefore, it may be confusing for many students to make informed decisions about which app to download. Providing empirical evidence and helping students identify whether the app is useful for them may be especially helpful. Teaching students how to identify a suitable evidence-based mental health app in the app store may also help in clearing uncertainty about whether a mental health app could actually be useful, which may be done through the *One Mind PsyberGuide* [37]. Interestingly, very few students were concerned about the costs associated with the app and access to technology in both our quantitative and qualitative findings. This suggests that students do feel they have sufficient resources to be able to access the app but are unsure of whether it would be useful or which they should select.

Qualitative findings around hesitancy suggested that many students simply felt that they did not need a mental health app or that it would not be effective. Although there are mental health apps that are effective, many mental health apps are not evidence based [38]. Therefore, students’ perceptions of mental health apps may be accurate. This also aligns with prior findings on mental health service use in college campuses. College students are often skeptical of the effectiveness of existing services [39]. Thus, hesitancy toward mental health services among college students may be a more pervasive issue that exists even outside the digital realm. Our qualitative findings suggest that there seems to be a preference toward face-to-face counseling, which also exhibits the assumption that using a mental health app would mean abandoning in-person therapy. Rather than considering mental health apps as a replacement for traditional forms of therapy, it should be noted that they may be used as adjuncts to care. Preference for face-to-face counseling and in-person interaction was the fourth major point of hesitation in using mental health apps, with about 1 in 8 students mentioning this preference. It may be useful for campuses to market mental health apps as additional resources rather than as a replacement for traditional forms of therapy. Although campuses do not directly suggest that an app should be a replacement, it may be helpful to reiterate to students that the app should be used in addition to other resources to combat the misconceptions that many students have. Furthermore, students often experience long wait times and issues with scheduling appointments for college counseling services [5]. These apps could be useful for students while waiting for counseling services, as mental health apps can be accessed immediately.

### Limitations

As previously mentioned in *Methods* section, demographic characteristics of the elective module sample and overall sample tended to be relatively similar. However, the elective module participants had a larger percentage of students pursuing higher levels of education. More students in the elective module were pursuing graduate studies. Furthermore, more students were pursuing a bachelor’s degree in the elective module, while a smaller percentage were pursuing an associate’s degree when compared with the overall sample. We noted earlier that app adoption may differ among types of college or university settings and differences in adoption rates might also exist among types of degrees. In addition, issues such as accessibility of resources could differ in the overall sample compared with the elective module, as community college students often experience severe psychological concerns with less institutional support for mental health resources [40]. With fewer students in the elective module pursuing an associate’s degree, there remains uncertainty about whether students in a 2-year university may have had different attitudes toward mental health apps.

It should be noted that the pandemic may have affected this study and attitudes toward mental health apps. Some students might have provided data after the March 2020 shutdown, which had an impact on their mental health, campus resources, and broader aspects of student life. The data set did not provide the survey completion dates for each participant; therefore, we were unable to exclude those who completed the survey after March 2020 or compare results across those who completed before and after March 2020. However, it is difficult to predict the long-term impact of the pandemic, as it was shaping people’s knowledge and interest in using digital resources to support their mental health [41]. Certainly, college and university campuses are reconsidering opportunities for remote and hybrid services for a variety of purposes, including mental health care.

### Conclusions

Our findings reflect the need for college campuses to provide resources tailored to the needs of students. Campuses should not only provide mental health apps as a resource but also provide resources that clear uncertainty regarding available apps. It is essential to consider why people use or do not use mental health apps and what is needed, in addition to the apps to support their use. On the basis of the responses of the students, plenty of hesitation continues to inhibit app adoption, with many students experiencing uncertainty about using a mental health app. In students who chose to use a mental health app, the presence of anxiety seemed to predict app adoption. As students tend to begin using mental health apps in colleges, it has become imperative to provide the resources necessary to promote mental well-being on campus.

## Abbreviations

GAD-7: Generalized Anxiety Disorder Scale-7
PHQ-9: Patient Health Questionnaire-9
